# Measuring the effects of laboratory automation: The power of empirically derived models

**DOI:** 10.1155/S1463924692000130

**Published:** 1992

**Authors:** Gerry Fitzgerald, Jim Swanson

**Affiliations:** SmithKline Beecham R&D 709 Swedeland Road, M/S L-331 King of Prussia Pennsylvania USA

## Abstract

Laboratory data systems are automated for a variety of scientific and management reasons. A key part to maintaining these systems is to regularly assess the impact that automation has had on the laboratory and the organization as a whole. Smith Kline Beecham R&D is using a number of different types of measurement, as well as a number of different tools, for measuring how automated laboratory systems are affecting the workflow and information flow in the laboratory. This targeted programme of metrics has increased management confidence in laboratory automation efforts, helped anticipate data processing bottlenecks, and highlighted end-user support needs.


Journal of Automatic Chemistry, Vol. 14, No. 2 (March–April 1992), pp. 55–57

### Measuring the effects of laboratory automation: The power of empirically derived models

**Gerry Fitzgerald and Jim Swanson**
*SmithKline Beecham R&D, 709 Swedeland Road, M/S L-331, King of Prussia, Pennsylvania, USA*

*Laboratory data systems are automated for a variety of scientific and management reasons. A key part to maintaining these systems is to regularly assess the impact that automation has had on the laboratory and the organization as a whole. SmithKline Beecham R&D is using a number of different types of measurement, as well as a number of different tools, for measuring how automated laboratory systems are affecting the workflow and information flow in the laboratory. This targeted programme of metrics has increased management confidence in laboratory automation efforts, helped anticipate data processing bottlenecks, and highlighted end-user support needs.*

### Introduction

SmithKline Beecham operates a complex ensemble of laboratory automation applications. These applications have been designed and implemented through a variety of technological eras and for a wide variety of scientific and business objectives. The organization needs constantly to determine whether the current systems are the optimal methods for meeting the needs of R&D, or if new systems are required. It needs to be understood that the automation of a laboratory fundamentally changes the operation of the laboratory, often in unpredictable ways. Allocation of limited laboratory automation resources also demands that opportunities for improved automation are identified.

The company's users are quite technically sophisticated. However, their area of expertise is biology, biochemistry, or other areas of biotechnology—only a few have (or need) a sophisticated understanding of how their computer systems work. They may not notice error messages, or may blame poor system performance on general conditions, rather than on faults that have crept into their particular applications. Alternately, some users may be 'pushing' systems beyond their designs out of need for a new system, and inadvertently introduce problems that were not anticipated. By monitoring applications in a standard way, application problems can be separated from system problems.

Few people argue about the need to monitor applications, measure performance, and report key indicators to management. The authors hope to go beyond those 'motherhood and apple pie' pronouncements and propose a detailed strategy for an automated laboratory. The key questions asked by most laboratory managers asked to introduce metrics are

(1) What parameters to measure?

(2) How to measure those parameters?

(3) How to report the information gathered?

The authors' starting point is 'Why do you need metrics?'

### Experimental

SmithKline Beecham's quest for a targeted, effective system of laboratory automation metrics began with surveys and interviews of senior management. They were to be the primary audience for the information from the system, so it was only natural to ask what information they would be seeking. After several iterations of 'blue sky' and 'hard realities', a short list of six key objectives that the metrics system should work toward was obtained. These were:

(1) *Capacity planning*—when to purchase new capital equipment (instruments, computers and peripherals), and in what amounts, to meet anticipated needs

(2) *Cost justification*—proving the cost/benefit justifications for the original systems to be accurate (or even conservative)

(3) *Allocation of resources*—which laboratory automation systems had the most potential for improvement?

(4) *Performance improvement*—what could be done to make the performance (uptime/response time) of existing systems more predictable?

(5) *Job satisfaction*—what could be done to show the people who implemented the system (often with considerable effort) that their work had paid off?

(6) *Process improvement*—what opportunities still existed for improving the workflow in the laboratory, and what opportunities existed in improving the service level provided to users of the system?

The next step was to determine how to generate measurements that would meet these objectives. The authors were also limited by rather severe resource constraints for this project, as it was designated as 'overhead to overhead'. Measurements had to be non-intrusive on the existing systems, and overall all of our metrics were to use no more than 2% of the available CPU. Information had to be presented in simple terms, avoiding computer jargon. The authors also determined to automate the measurement and analysis process, as manually producing such information would not improve our job satisfaction. Two advantages turned out to be

© Zymark Corporation 1992

vital. One was that it was not necessary to be overly precise in the measurements, as resources were allocated and purchased in large chunks in relation to the resource usage of the laboratory automation systems. The other advantage was that it was possible to focus on just a few issues, rather than trying to measure everything that could be measured.

The first set of metrics was aimed at measuring capital resource use. It was necessary to know for each application, what the total use of CPU, disk, and memory was, so changes could be translated into budget estimates. Operating system utilities existed to aid in gathering this information. The capital resource use was related to the volume of work (samples/transactions) processed during the same time period. This would, over time, generate curves showing how resource use varied with volume of work, a key measurement for capacity planning.

Allocations to each application for maintenance costs for the software licences and hardware were added to this. This became the financial cost of ownership. The support effort attributed to the application also had to be measured, and its cost added to the financial cost to obtain a total cost of ownership.

The benefits of the application would be measured in terms of the volume of data, samples, or transactions processed through the system. These benefits would be related to the performance of the system. The slower the system became (as data accumulated or as the workload of the laboratory increased), the less incremental benefit the system would have. Part of that performance rating was also to be based on the effectiveness of support. Penalties for downtime, errors, and long response time to support questions would decrease the benefit of the system.

Of course, the true financial impact of these things would be very hard to determine. However, by presenting the information in units clear to the laboratory (samples processed, hours connected, dollars of maintenance paid, and maintenance hours required), managers could use their own estimates of the value of these things to arrive at their decisions.

The project was begun with a set of prototype applications. Rather than measure every application, the most important applications in each category were chosen and used as proxies for the whole task. RS-232 data collection, chromatography, data analysis tools, and general utilities were evaluated. Proxies were RIGEL (an in-house RS-232 data collection system), PE Nelson's ACCESS*-CHROM, RS/1 and VAX Mail. Coverage was gradually expanded as the data collection and reporting tools were developed.

### Results and discussion

The prototype applications provided excellent illustrations of the methods for acquiring and reporting metrics information. Data were collected both continuously and in discrete chunks. All of the data were aggregated and reported on a monthly basis. The goal set was to report the data no later than the third working day following the close of the month. In some case minor modifications had to be made to applications in order to automatically count the number of samples, users or translations. In most cases, simple once per month procedures could gather the required information, process it, and print and mail the reports.

For example, the RIGEL application required a variety of measurement techniques. First, the application was modified to log usage by each user and by each instrument collecting data. The application would also log the number of samples in each run. A separate class of problems was created within the help desk problem tracking system to record RIGEL specific calls. (Previously all laboratory automation calls had been lumped together.)

The chromatography data had to be gathered without modifying the application, as this was a purchased, rather than a developed package. The number of RAW data files produced by the system was used as a proxy of the number of injections on the system. A method of scanning the RAW data files to track the instruments used was devised and discarded, as it took too long to run. Total disk usage of the RAW, processed, and method files was measured by looking at the creation dates of the files. Performance and CPU load were measured by re-integrating a standard run of 100 run files. The load of data collection was assumed to be negligible. Best results were obtained after we copied the files before re-integrating, to mimic the current level of disk fragmentation.

RS/1 usage was trickier to measure. A batch procedure was used to scan the users logged into the system. The batch procedure used a DECUS utility, WHOZON, that would display the image name that the user was running. (The procedure must be installed with privileges.) The number of users running the RS/1 image was logged to a file every 15 minutes by the batch procedure. Performance was measured by running a short standard job that generated a standard curve and plotted a set of data every 60 minutes during prime time and recording the system resources used. Similar procedures were used for the mail utility. The batch procedure has now been expanded to simultaneously monitor over 50 utilities to date.

The reports generated from this information had to be carefully crafted to present the information in a suitably summarized fashion. A flexible set of reports was chosen. Managers could request any subset of the reports, depending on the applications of interest to their groups and their responsibilities for those applications (throughput, capital improvement, support). Every month, a two paragraph summary was produced of the metrics, modelled on a weather report. This summary is included in the departmental monthly report circulated to senior management.

Graphical trend reports have been the best received. To smooth out normal, short-term variations in workload, moving averages are employed. These trends allow senior managers to predict when capital equipment will be needed. They have often been quite surprised at the volume of samples, transactions, data, etc., that flows through the laboratory. Milestones are highlighted for the managers, such as '50% increase in sample throughput since the system was installed', so that they may convey congratulations to the laboratory. Unanticipated decreases in system use or throughput usually bring a visit from an analyst to find out if the workload is down or the system is not meeting users' needs.

One particular report on the utility software has saved us quite a bit of money. By analysing the number of simultaneous users for a particular utility, it is possible to negotiate improved, and less expensive licensing agreements with most of the vendors. These licence agreements also give increased flexibility in distributing the application to multiple platforms. The monitoring on RS/1, for example, shows a continued decline in use. This corresponds to a major campaign of putting PCs into the laboratories. Anecdotal evidence points to the replacement of the package by PC-based spreadsheets and graphics packages.

Another very useful set of reports have been the support reports. These reports show the number and types of calls handled by the support analysts, and the amount of time these analysts spent responding to those calls. Each application has between five and a dozen call types, which are general types of questions or problems with that application. For example, in data acquisition application, the problem type can be a communications problem, a parsing problem, a documentation problem, or even a user training problem. These problems can be accumulated over all data acquisition applications to give an overall view of communications reliability, documentation quality, and user training needs. The conclusions are submitted to senior management, along with the costs (in person-hours) that could be avoided.

### Successes

The models that are used for the various applications that the authors are tracking have led to a variety of successes. These successes have manifested themselves as tools, guidelines, and actions, not only for the applications we are currently tracking, but also affecting areas such as resources, finances, and job satisfaction.

The information gathered from a particular application gives a good overview about usage, performance, and support requirements. Statistical analysis of the metrics yields information concerning processor constraints such as CPU and I/O load, licence requirements for multi-user products, such as RS/1, and capital needs from current trends. The performance expectations of the user community have been more proactively met due to anticipating such resource requirements as computing power, people and finances. Support of these applications has also been enhanced in such areas as error tracking improved response time, and coordinated support.

The benefits of these models has allowed for guidelines to be established for future applications that are either developed or purchased. These guidelines include the ability to report on usage and performance statistics and information about the application to perform proper problem tracking. As these guidelines are implemented the burden of support, resource allocation, and financial requirements becomes less of a best-guess approach and more of an educated decision.

The models have also yielded successes in financial savings and overall job satisfaction. As resources and licences are more accurately allocated and throughput increased due to error reduction, financial analyst resources have been able to be better utilized. Thus allowing for more projects to be financed, better use of available personnel to complete more projects, and overall greater job satisfaction due to increased productivity.

### The future

The metrics that are implemented are applied to a small subset of the total number of applications that are currently in place. Since the authors' role as developers and support analysts exists beyond the abbreviated list of currently tracked applications and systems it is essential that we broaden our scope. The benefits that we have achieved thus far can be extended to other areas such as PCs. All new applications now incorporate metrics in the design phase and into support mechanisms.

The effort required to broaden our scope will require us to educate senior management on the benefits of metrics. This is required to demonstrate the direct financial benefits of incorporating metrics within applications. The time spent developing the metrics and tracking the applications has been time well spent. The benefits have greatly outweighed the effort involved.

